# Chronotype of pregnant women with diabetes mellitus: what is the relationship with maternal and fetal outcomes?

**DOI:** 10.20945/2359-4292-2023-0071

**Published:** 2024-02-02

**Authors:** Johnnatas Lopes, Julio Martinez Santos, Guilherme Ribeiro, Matheus Queiroz, Jorge Fernando Pereira Silva

**Affiliations:** 1 Universidade Federal do Vale do São Francisco Petrolina PE Brasil Universidade Federal do Vale do São Francisco, Petrolina, PE, Brasil

**DEAR EDITOR**

Gestational diabetes seems to be related to the modified circadian rhythm in this period (1). However, further clarification is needed with covariance adjustments for the variables involved and longitudinal design. Facanha and cols. (2) proposed a research strategy in order to answer some questions of scientific interest, such as the relationship between morningness/eveningness chronotypes on maternal and fetal outcomes. However, we identified gaps that could be better clarified by the authors in measuring these relationships.

In the study, Tables 1 to 3 report the differences between pregnant with diabetes mellitus in terms of different factors depending on the morningness/eveningness chronotype. In maternal outcomes, a difference was identified in pre-eclampsia and in neonatal patients in intensive care and, marginally, in prematurity. This information only tells us that there is a difference between the groups and that it does not exclude the need to test the relationship of maternal factors with their outcomes and also fetal outcomes in regression models (3).

However, Facanha and cols. (2) presented only the unadjusted model for some factors, apparently those that revealed differences between chronotypes, when in fact they should be the factors theoretically related to the outcome of pre-eclampsia, such as demographic and clinical variables and comorbidities. These regressions go beyond differences between groups, it allows for control of covariance in the raw model (3).

In addition, there is a mistake in the selection of independent variables in the model presented for the pre-eclampsia outcome. The age variable included in the adjusted model should not be used because it did not reach the minimum criterion of p < 0.20 in the crude analysis. This can interfere with the quality and usefulness of the model. It would also be of great value to develop the model for neonatal outcomes.

Therefore, we understand as relevant the construction of a hierarchical model for neonatal outcomes and a better exploration of the independent variables in the elaboration of the model, the outcome such as pre-eclampsia in order to exclude or not the relation with the chronotype of the pregnant woman with diabetes mellitus.

## References

[1] Weschenfelder F, Lohse K, Lehmann T, Schleußner E, Groten T (2021). Circadian rhythm and gestational diabetes: working conditions, sleeping habits and lifestyle influence insulin dependency during pregnancy. Acta Diabetol.

[2] Facanha CFS, Alencar VS, Machado PS, Macêdo RBL, de Bruin PFC, Costa E (2023). Morningness/eveningness in gestational diabetes mellitus: clinical characteristics and maternal-neonatal outcomes. Arch Endocrinol Metab.

[3] Suárez E, Pérez CM, Rivera R, Martínez MN (2017). Poisson Regression Models for Cohort Studies. In Applications of Regression Models in Epidemiology.

